# Determining a Clinically Applicable Cutoff in AI Algorithms for Predicting Clinical Deterioration: A Workload-Constrained, Alarm-Based Approach

**DOI:** 10.3390/jcm15145753

**Published:** 2026-07-22

**Authors:** Jaewon Jang, Yong Jun Choi, Taeyong Sim, Ki-Byung Lee, Ji-Hyun Kim, Eun Young Cho, Yuhyun Choi, Sungsoo Hong, Bo Mi Jung, Soo-Jeong Kim, Won Gi Hong, Jae Hwa Cho

**Affiliations:** 1 AITRICS Corp, Seoul 06221, Republic of Korea; jjw2582@aitrics.com (J.J.); tae@aitrics.com (T.S.); jhkim@aitrics.com (J.-H.K.); ey10.cho@aitrics.com (E.Y.C.); burnham@aitrics.com (Y.C.); sshong@aitrics.com (S.H.); 2 Division of Pulmonary and Critical Care Medicine, Department of Internal Medicine, Gangnam Severance Hospital, Yonsei University College of Medicine, Seoul 04763, Republic of Korea; cyj0717@yuhs.ac (Y.J.C.); jbm322@yuhs.ac (B.M.J.); 3 AITRICS Inc., McLean, VA 22102, USA; 4 Division of Pulmonary, Allergy and Critical Care Medicine, Department of Internal Medicine, Chuncheon Sacred Heart Hospital, Hallym University Medical Center, Chuncheon 24253, Republic of Korea; hallas79@hallym.or.kr; 5 Department of Internal Medicine, Yongin Severance Hospital, Yonsei University College of Medicine, Yongin 16995, Republic of Korea; alvin97@yuhs.ac; 6 Department of Hospital Medicine, Yongin Severance Hospital, Yonsei University College of Medicine, Yongin 16995, Republic of Korea

**Keywords:** in-hospital adverse events, clinical deterioration, deep learning, clinical decision support system, early prediction

## Abstract

**Background**: This retrospective study introduces an AI-driven VitalCare-Major Adverse Event Score (VC-MAES) developed to predict major in-hospital adverse events and determine optimal cutoff thresholds. VC-MAES was originally developed to predict a composite outcome including unplanned intensive care unit (ICU) transfer, in-hospital cardiac arrest, and mortality; the present study evaluates its performance for the composite of unplanned ICU transfer and in-hospital cardiac arrest. **Methods**: Patients aged ≥19 years in Yongin Severance Hospital between 1 March 2020, and 31 December 2022, were included. The primary outcome was clinica l deterioration, defined as unplanned ICU transfer or in-hospital cardiac arrest. Secondary outcomes included model performance metrics (area under the receiver operating characteristic (ROC) curve, sensitivity, specificity, F1 score) and optimal alarm frequency (number of alarms per 100 patient-days) across cutoff determination methods (Youden’s index, F1 score, Euclidean distance, and alarm-based approach); they were used to determine optimal cutoff values. 20 July 2026. **Results**: VC-MAES achieved an area under the ROC curve of 0.895(full evaluable sample) at 6-h intervals, outperforming traditional early warning systems. It exhibited improved sensitivity (0.5853) compared to NEWS (0.3775) and MEWS (0.3049) while maintaining a specificity exceeding 0.95, balancing alarm frequency and predictive accuracy (all figures based on each model’s own full evaluable sample: VC-MAES, n = 7425 events; NEWS, n = 3494; MEWS, n = 3198). On the common prediction time-point sample (n = 1678 events), the alarm-based cutoff yielded a sensitivity of 0.5244 for VC-MAES versus 0.2271 for NEWS and 0.1740 for MEWS, with alarm frequencies of 9.67, 7.07, and 5.58 per 100 patient-days, respectively. When compared on a common set of prediction time-points, VC-MAES (AUROC 0.832 on the common sample, 95% CI: 0.822–0.842) significantly outperformed NEWS (AUROC 0.743, 95% CI: 0.732–0.756; DeLong Z = −14.19, *p* < 0.0001) and MEWS (AUROC 0.706, 95% CI: 0.693–0.720; DeLong Z = −18.22, *p* < 0.0001). ROC-based methods, such as Youden’s index and Euclidean distance, were deemed unsuitable for practical clinical use. **Conclusions**: Conventional ROC-based cutoff selection methods may not be suitable for real-world clinical implementation due to excessive alarm burden. An alarm-based cutoff approach provides a more clinically applicable strategy by balancing predictive performance with manageable alarm frequency. These findings highlight the importance of incorporating clinical usability into cutoff determination for AI-based early warning systems.

## 1. Introduction

The early detection of acute deterioration in hospitalized patients is crucial for improving outcomes and reducing preventable adverse events. Traditional early warning scores (EWS), such as the National Early Warning Score (NEWS) and Modified Early Warning Score (MEWS), rely primarily on basic vital signs and mental status assessments, making them simple and practical for clinical implementation [1]. However, they incorporate a limited range of data and may overlook important factors such as laboratory results and comorbidities, potentially reducing predictive accuracy in certain cases [2,3].

With the widespread adoption of electronic medical records (EMRs) and the accumulation of large, complex datasets, deep-learning–based artificial intelligence (AI) has emerged to handle inputs that exceed the capabilities of simple rule-based algorithms. Despite the growing adoption of AI and Machine Learning (ML) models in clinical practice, there is no clear consensus on the optimal methods for determining the cutoff values to trigger clinical interventions. Statistically, the optimal cutoff values are determined using methods such as Youden’s index, which identifies the point on the receiver operating characteristic (ROC) curve that maximizes the combined sensitivity and specificity. However, the direct application of these statistically determined optimal cutoff values in clinical practice is limited because of constraints related to human resources, time, and cost. In clinical scoring systems, such as EWS, these cutoff values are typically determined by carefully balancing sensitivity and specificity to ensure an effective response while minimizing false alarms. However, the most efficient and clinically meaningful treatment approach for AI-based systems remains unclear.

We developed the VitalCare-Major Adverse Event Score (VC-MAES), a deep learning-based clinical decision support system designed to predict in-hospital adverse events—namely, unplanned intensive care unit (ICU) transfers and cardiac arrests—in patients admitted to general wards. VC-MAES continuously generates a Major Adverse Event Score (MAES) in real time by analyzing structured data extracted from EMRs.

This study evaluates the practicality of using VC-MAES for predicting clinical deterioration and compares different methods for establishing cutoff values in a real-world clinical setting.

## 2. Materials

### 2.1. Study Design and Setting

We conducted a retrospective study at the Yonsei University Yongin Severance Hospital, a 708-bed medical center in Republic of Korea. We included patient admission data collected from 1 March 2020, to 31 December 2022. The primary objective was to compare cutoff values derived from ROC curves, F1 scores, and alarm frequency (alarms per 100 patient-days) and to assess their clinical implications. Using the metric of the total number of alarms per 100 patient-days provides a standardized method for comparing alarm data across different healthcare settings [4,5,6].

### 2.2. Patient Selection and Data Collection

**Figure 1:**
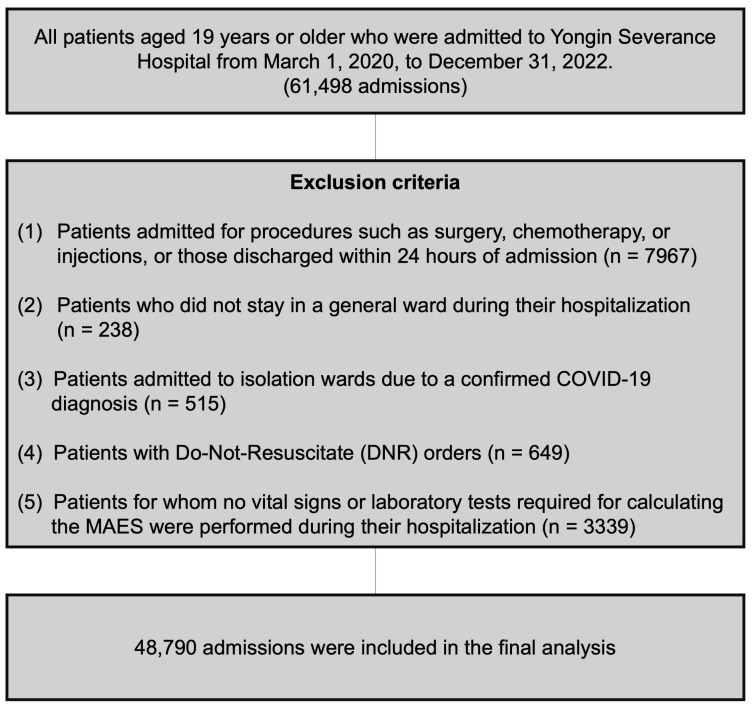
Data flowchart.

All patients aged 19 and above admitted to Yongin Severance Hospital between 1 March 2020–31 December 2022 were initially included in the study. Exclusion criteria were as follows: Patients admitted solely for procedures such as surgery, chemotherapy, or injections and subsequently discharged within 24 h; patients who did not stay in a general ward during hospitalization; patients with a confirmed COVID-19 diagnosis; patients without any vital signs or no laboratory tests required for MAES calculation; and patients with Do-Not-Resuscitate (DNR) orders (Figure 1). We extracted demographic data, vital signs (systolic and diastolic blood pressure, heart rate, respiratory rate, body temperature, and oxygen saturation), laboratory results (bilirubin, lactate, pH, sodium, potassium, creatinine, hematocrit, white cell count, bicarbonate, platelet count, and C-reactive protein (CRP)), and outcomes (ICU admissions, cardiac arrests, and mortality) from the EMR system using the Severance clinical research analysis portal (SCRAP 2.0).

### 2.3. VC-MAES Algorithm

VC-MAES is an AI model based on deep learning designed to predict adverse events in hospitalized patients. The model was trained on data from 334,185 hospital admissions involving 209,825 adult patients (aged >19 years) treated between 2013 and 2017 at Yonsei Severance Hospital, a 2454-bed tertiary academic center in Seoul, South Korea. The dataset covered over 35 medical and surgical subspecialties. VC-MAES predicts major adverse events within the next 6 h by generating a score from 0 to 100, with higher scores indicating greater risk. The model primarily uses five vital signs (systolic and diastolic blood pressure, heart rate, respiratory rate, and body temperature) and age. However, this model can incorporate additional variables, including oxygen saturation, the Glasgow Coma Scale, and specific laboratory results (bilirubin, lactate, pH, sodium, potassium, creatinine, hematocrit, white blood cell count, bicarbonate, platelet count, and CRP) if available [7,8].

The VC-MAES model used in this study was version 1.4.1, the clinically deployed version in routine operation at Yongin Severance Hospital during the study period. The model weights and scoring logic were locked before the evaluation period began; no updates or retraining occurred during the study period. In the general ward setting, clinical variables are measured at irregular and relatively infrequent intervals (e.g., vital signs typically every 4–8 h; laboratory tests ordered on clinical indication). Under these conditions, a last-observation-carried-forward (LOCF) approach was applied for missing vital signs within a 24-h lookback window, reflecting the clinically reasonable assumption that a patient’s physiological state remains relatively stable between consecutive measurements and that the most recent observation represents the best available estimate of the current value until the next measurement is obtained. For laboratory variables with no prior observation available, population-based normal reference values were used as initial values, consistent with the model’s original training protocol. Variable-specific missingness proportions and mean measurement intervals for all predictor variables are reported in Supplementary Table S5.

### 2.4. Clinical Outcomes

The primary outcomes were in-hospital cardiac arrest (IHCA)—defined as a loss of circulation requiring resuscitation efforts such as chest compressions, defibrillation, or both—and unplanned ICU transfer (UIT), defined as ICU admission owing to unexpected clinical deterioration in patients from general wards rather than from the operating room or emergency department. We defined the occurrence of IHCA or UIT as clinical deterioration events (CDEs).

### 2.5. Performance Evaluation and Statistical Analysis

We compared demographic and clinical characteristics between the control and event groups. Categorical variables were presented as frequencies (percentages). Continuous variables were presented as mean ± standard deviation for normally distributed variables and median (interquartile range (IQR)) for non-normally distributed variables. Normality assumptions for continuous variables were confirmed using the Shapiro–Wilk test.

The accuracy of the model in predicting adverse events was assessed using the area under the ROC curve (AUROC). Sensitivity and specificity were calculated at multiple thresholds to determine the optimal cutoff point. Several statistical methods used to determine the optimal cutoff value, including Youden’s index, F1 score, Index of Union (IU), and Euclidean distance (ED), were evaluated. Additionally, we evaluated alarm-based optimal cutoff values within the optimal alarm range of 3–10 alarms per 100 patient-days [4,5,6]. For each model, we calculated the total number of alarms per 100 patient-days across score values and set the cutoff value within a clinically optimal alarm count range to establish an alarm-based cutoff value. Statistical significance was determined at a *p*-value threshold of 0.05. All statistical computations were executed in Python, version 3.11.5. We used the pROC package in R (version 4.4.2) for ROC curve analysis. Artificial intelligence tools were not used for data analysis, statistical analysis, or interpretation; AI-assisted language editing is described in the Acknowledgements. Patients with multiple admissions during the study period were retained, and each admission was treated as an independent observation; confidence intervals were estimated using patient-level cluster bootstrap resampling to account for within-patient correlation across admissions. The unit of analysis was the individual observation time-point, with each admission contributing multiple correlated measurements. To account for within-admission clustering, all 95% confidence intervals were calculated using patient-level cluster bootstrap resampling (1000 iterations), in which all observations from the same patient were resampled together. An alarm was defined as any patient-day on which the model score exceeded the cutoff threshold at least once. Multiple threshold crossings within the same patient-day were counted as a single alarm. Alarm frequency was expressed as the number of alarm patient-days divided by total patient-days, multiplied by 100. This per-patient-day counting convention was applied consistently across all models and thresholds. For each CDE, the model score at exactly 6, 8, 12, and 24 h prior to the event was extracted (or the nearest available time-point within a ±30-min window). For non-event admissions, a random index time was sampled from within each admission to construct a matched observation set with a comparable distribution of time-within-admission. For NEWS and MEWS, input variables were obtained from the most recent available measurements within a 4-h lookback window at each index time-point; oxygen supplementation status and level of consciousness were taken from the most recent nursing documentation within the same window. For the formal head-to-head comparison of discriminative performance, analyses were restricted to time-points at which all three scores (VC-MAES, NEWS, and MEWS) could be simultaneously computed (i.e., where all required inputs were available for each model); this subset is referred to as the common prediction time-point sample. To enable direct comparison of operating-point metrics across models, alarm-based cutoff performance (sensitivity, specificity, PPV, and alarm frequency) was additionally computed on this common prediction time-point sample (674,560 time-points; 1678 events; VC-MAES full sample: 7425 events; NEWS full sample: 3494 events; MEWS full sample: 3198 events).

### 2.6. Ethics

This study was approved by the Institutional Review Board of the Yongin Severance Hospital (Approval No. 9-2023-0048). The requirement for obtaining informed consent was waived by the IRB owing to the retrospective nature of the study using de-identified clinical data. All procedures were performed in accordance with the ethical standards of the institutional and national research committees and with the Helsinki Declaration and its later amendments. This study complies with all applicable institutional and national regulations and ethical guidelines. A copy of the IRB approval certificate (with English translation) and the completed Human Participant Declaration Form have been submitted to the journal for verification.

## 3. Results

### 3.1. Baseline Characteristics

Over the 34-month study period, we included 48,790 admissions and analyzed 3,067,883 time-point entries. Table 1 presents the baseline characteristics of the study population. We observed 182 IHCA and 524 cases of UIT, corresponding to 2.9 IHCA and 8.5 UIT cases per 1000 admissions among all hospitalized patients. Admissions associated with CDEs occurred in older and more frequently male patients, and were more often medical (versus surgical) admissions.

We observed significant differences in most components of the MAES, particularly in the median complete blood count results for each patient during hospitalization: CDE patients had lower hematocrit levels (27.9 [25.8–31.6] vs. 35.9 [31.6–39.9]), higher WBC counts (9900 [7300–12,900] vs. 7300 [5700–9300]), and lower platelet counts (170,500 [96,500–239,000] vs. 222,000 [177,000–272,000]; Table 1), with all comparisons showing *p* < 0.001. The median CRP levels during hospitalization were higher in CDE patients than in non-CDE patients (46.9 [21.4–83.5] vs. 8.3 [1.3–33.7], *p* < 0.001).

The minimum, maximum, and median MAES, NEWS, and MEWS scores during hospitalization were all significantly higher among CDE patients (Table 1).

### 3.2. Predictive Performance of MAES for CDE

**Figure 2:**
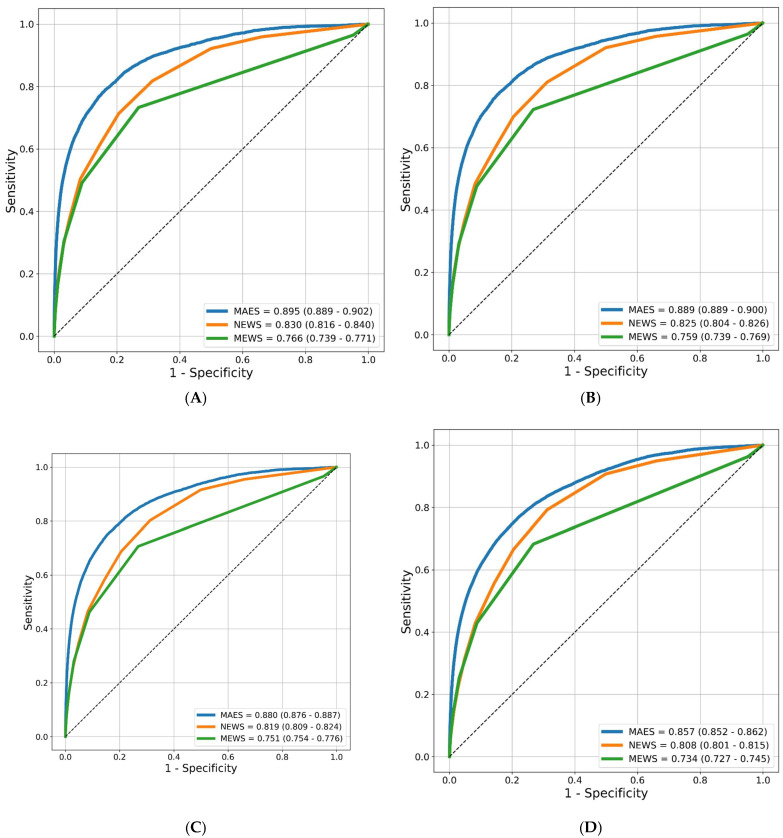
Comparison of predictive performance between VC-MAES, NEWS, and MEWS across multiple prediction horizons. Receiver operating characteristic (ROC) curves for predicting clinical deterioration at (**A**) 6 h, (**B**) 8 h, (**C**) 12 h, and (**D**) 24 h prior to event occurrence. The dashed diagonal line represents the no-discrimination reference line. VC-MAES consistently outperformed conventional early warning scores across all prediction horizons, particularly at shorter prediction intervals, indicating improved early detection of clinical deterioration.

The predictive performances of MEWS, NEWS, and VC-MAES were compared at prediction intervals of 6, 8, 12, and 24 h before CDE (Figure 2A–D). In MAES, NEWS, and MEWS, the AUROC values increased as the time approached the CDE within 24 h, with MAES consistently displaying the highest AUROC, followed by NEWS and MEWS at each time interval (Table 2). The MAES model achieved the highest performance in predicting events 6 h before CDE occurrence, with an AUROC of 0.895 (full evaluable sample; 95% CI: 0.889–0.902) (Figure 2A). To formally compare discriminative performance, DeLong tests were performed on the common evaluable sample—defined as time-points at which all three scores (VC-MAES, NEWS, and MEWS) could be simultaneously computed. VC-MAES (AUROC 0.832, 95% CI: 0.822–0.842) significantly outperformed NEWS (AUROC 0.743, 95% CI: 0.732–0.756; Z = −14.19, *p* < 0.0001) and MEWS (AUROC 0.706, 95% CI: 0.693–0.720; Z = −18.22, *p* < 0.0001) at the 6-h horizon, with consistent superiority across all prediction horizons (all *p* < 0.0001; Table 2). Given the low event prevalence (~1.4%), the area under the precision-recall curve (AUPRC) was additionally calculated using patient-level bootstrap resampling. At the 6-h horizon, patient-level AUPRC values were 0.0532 (95% CI: 0.0403–0.0694) for VC-MAES, 0.0093 (0.0074–0.0120) for NEWS, and 0.0084 (0.0065–0.0113) for MEWS, reflecting the superior precision-recall trade-off of VC-MAES in this imbalanced setting. Full AUROC and AUPRC results across all prediction horizons are provided in Supplementary Table S4. A calibration plot for VC-MAES at the 6-h horizon is presented in Supplementary Figure S1.

### 3.3. Comparisons of Optimal Cutoff Values in MAES

In Youden’s index analysis, the optimal cutoff value for MAES was determined to be 15, achieving a sensitivity of 0.7825 and specificity of 0.8429. This resulted in an average of 19.2 alarms per 100 patient-days at this cutoff value. Table 2 shows that the cutoff value at the Index of Union (IU) was the same as for Youden’s index. Additionally, the cutoff value at the minimum ED was 14, with a sensitivity of 0.7969 and specificity of 0.8272, generating 21.1 alarms per 100 patient-days. The F1 score reached its maximum at a cutoff value of 60, with a sensitivity of 0.1938 and specificity of 0.9970, generating 0.7 alarms per 100 patient-days.

### 3.4. Comparison of Alarm-Based Optimal Cutoff Values Between MEWS, NEWS, and MAES

**Figure 3:**
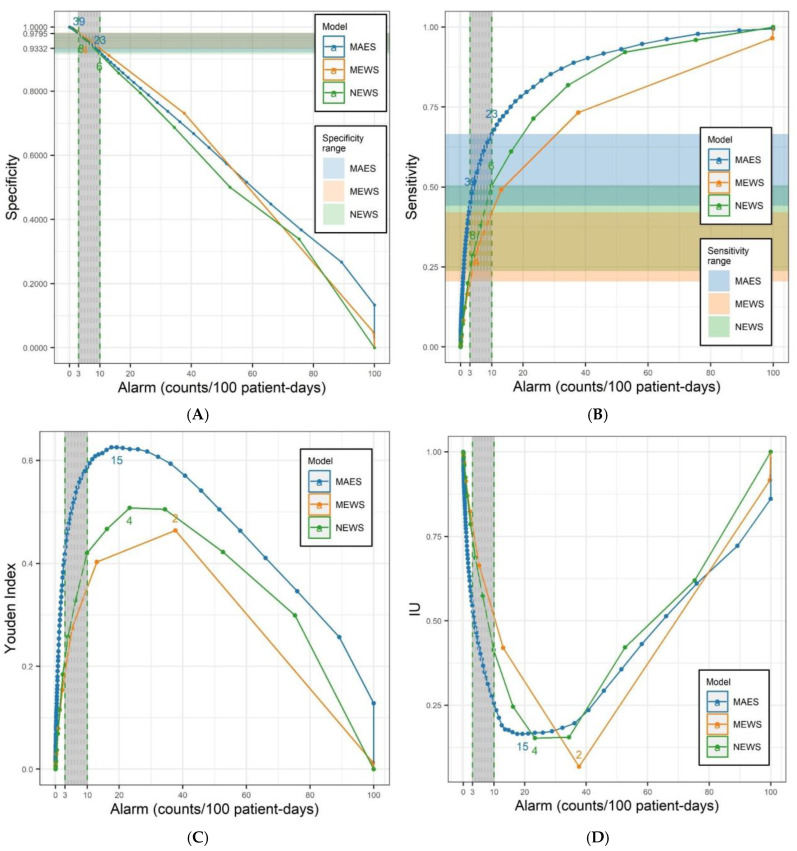

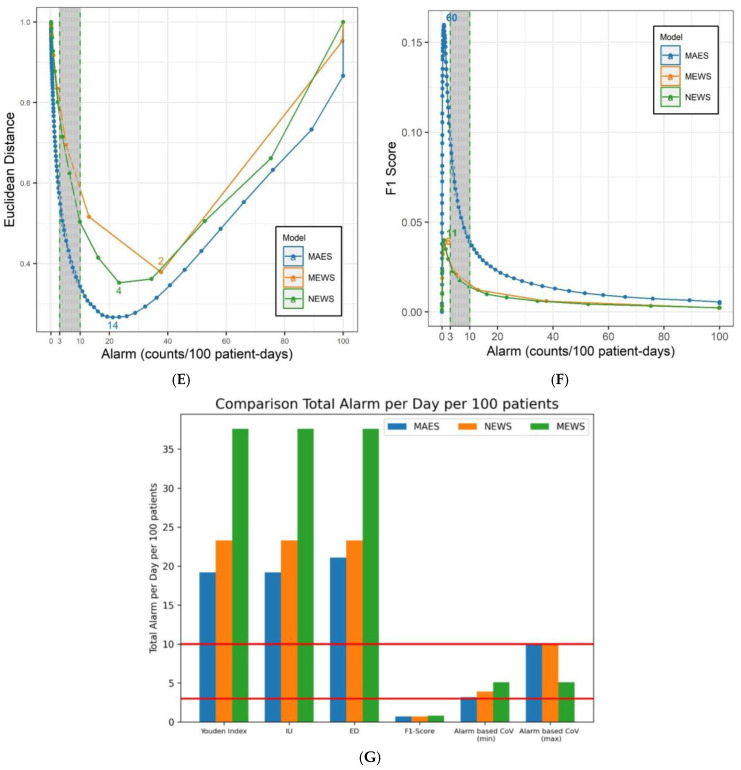
Evaluation of cutoff selection methods in relation to clinically acceptable alarm ranges. (**A**) Specificity across varying alarm counts for each model. (**B**) Sensitivity across varying alarm counts for each model. (**C**) Youden index values at different cutoff thresholds. (**D**) Index of union (IU) values across cutoff thresholds. (**E**) Euclidean distance (ED) across cutoff thresholds. (**F**) F1 scores across cutoff thresholds. (**G**) Total number of alarms per 100 patient-days across cutoff values. The gray shaded area indicates the clinically acceptable alarm range, corresponding to approximately 3–10 alarms per 100 patient-days in the present analysis. Numbers above each plot indicate the optimal cutoff values derived from each method. Traditional ROC-based methods result in alarm frequencies outside the clinically acceptable range, whereas alarm-based cutoff selection identifies thresholds that better align with clinical workflow.

We compared the frequency of alarms per day in 100 patients using MAES, NEWS, and MEWS. To adjust for differences in model scales, sensitivity, specificity, alarm frequency, and cutoff-related indices were analyzed assuming linear relationships between score scales and alarm-related metrics (Figure 3A–F). In all EWS models, the optimal cutoff values determined via traditional statistical methods did not meet the optimal alarm range (Figure 3G). For the MAES model, scores ranging from 23–39 generated 3.2–10.0 alarms per 100 patient-days. The cutoff values within the optimal alarm range for the MAES model showed specificity ranging from 0.9215–0.9812 (median 0.9514) and sensitivity ranging from 0.4412–0.6657 (median 0.5535). The cutoff value closest to the median and maximized sensitivity was 29. This value achieved a specificity of 0.9526 and sensitivity of 0.5853, and it generated 6.4 alarms per 100 patient-days (Figure 3A,B, and Supplementary Table S1). For the NEWS model, scores within the 6–8 point range generated between 3.9 and 9.9 alarms per 100 patient-days. Cutoff values within the optimal alarm range for NEWS showed specificity ranging from 0.9158–0.9795 (median 0.9477; Figure 3A) and sensitivity ranging from 0.2369–0.5053 (median 0.3711; Figure 3B). For MEWS, a score of 4 points generated 5.2 alarms per 100 patient-days. The specificity and sensitivity values corresponding to the optimal number of alarms per day in MEWS ranged from 0.9332–0.9831 (median 0.9582) and from 0.2045–0.4207 (median 0.3126), respectively (Figure 3A,B). The specificity range commonly applicable for optimal alarms across all models was 0.9332–0.9795 (median 0.9564; Figure 3A). The commonly used clinical cutoff points for NEWS and MEWS were also included within the alarm-based optimal cutoff value range [9,10,11].

### 3.5. Comparison of the Time Difference Between the First Prediction and CDE Occurrence Based on Optimal Cutoff Determination Methods

We compared the time difference between the first prediction (when the prediction model score exceeded the cutoff value) and the actual time of CDE occurrence across the MAES, NEWS, and MEWS models (Table 3). Using Youden’s Index and the IU, MAES, NEWS, and MEWS had average first prediction times of 255 min (95% CI: 254.5–257.1), 260.4 min (95% CI: 258.7–262.0), and 254.7 min (95% CI: 252.9–256.4), respectively. The ED results showed similar times, with MAES, NEWS, and MEWS at 258 min (95% CI: 257.1–259.6), 260.4 min (95% CI: 258.7–262.0), and 254.7 min (95% CI: 252.9–256.4), respectively. The F1 score yielded substantially shorter lead times before clinical deterioration events, with MAES, NEWS, and MEWS at 147 min (95% CI: 145.7–148.5), 221.4 min (95% CI: 219.6–223.2), and 202.5 min (95% CI: 200.4–204.5), respectively. When using the optimal alarm-based cutoff values, the average first prediction times were 219 min (95% CI: 218.3–221.2) for MAES, 241.5 min (95% CI: 239.7–243.3) for NEWS, and 223.4 min (95% CI: 221.4–225.3) for MEWS.

## 4. Discussion

This study evaluated the predictive performance of the MAES model against NEWS and MEWS in identifying CDEs in hospitalized patients. Our findings demonstrate that MAES consistently achieved higher AUROC values than NEWS and MEWS across all time intervals, with the peak performance occurring 6 h before CDEs. This suggests that MAES offers a more accurate and time-sensitive assessment of clinical deterioration risk, enabling earlier intervention and more efficient resource allocation. This study indicates that MAES outperformed traditional early warning systems, particularly at shorter prediction intervals. This finding suggests that MAES may facilitate earlier identification of patients at risk of deterioration, which could support more timely clinical intervention. Furthermore, we developed a clinically oriented cutoff-selection method that, although it introduces an approximate 30-min delay compared to statistically optimal thresholds, preserves an acceptable alarm frequency for predicting CDEs.

Reliance on AUROC and ROC-based methods for selecting cutoff values does not align well with clinical needs [2,3]. In settings where data are imbalanced—which is common when predicting rare events such as sudden deterioration—the AUROC can be misleading. To prevent alarm fatigue, clinicians require methods that balance sensitivity with a manageable number of actionable alarms. Alternative approaches should focus on clinically relevant metrics that consider an acceptable number of alarms and clinical context, ensuring that the model’s outputs integrate smoothly into the daily workflow of healthcare providers.

This study found that using ROC-curve-based methods, including Youden’s index, IU, and ED, for determining cutoff values is inefficient in predicting clinical deterioration in general wards. These methods may overwhelm staff with excessive alarms, causing them to miss critical events. Although the F1 scores balanced precision and recall, they did not necessarily align with clinical priorities. The sensitivity associated with the cutoff selected using the F1 score was low at 0.1938, with an average prediction time of 147 min, shorter than others. Additionally, the number of alarms was only 0.7 per 100 patient-days, making the criteria overly stringent (Table 2 and Table 3). Therefore, screening patients effectively and identifying true-positive cases becomes challenging.

Brajer et al., Capan et al., and Drew et al. reported that the optimal alarm cutoff range for EWS models, based solely on ensuring a clinically manageable workload to minimize false alarms, is 3–10 alerts per day per 100 admissions [4,5,6]. To facilitate comparison with this benchmark, alarm frequency in the present study was calculated on a per-patient-day basis (the number of alarm patient-days divided by total patient-days, multiplied by 100); this metric is conceptually analogous to the per-100-admissions-per-day unit when the analysis period spans the full admission. Therefore, when cutoff values were determined based on alarm counts, the results aligned more effectively with those of clinical practice [4,12,13,14,15,16,17,18]. In this study, the number of alarms generated by the predictive model was closely related to specificity, whereas sensitivity varied according to the model’s accuracy. Therefore, setting cutoff values based on a specificity threshold for an appropriate alarm rate appears reasonable. Optimal cutoff values determined using the alarm-based method for the three EWS models were achieved using the specificity threshold set between 93% and 98%. This strategy significantly reduced false positives and minimized alarm fatigue among clinical staff. In this framework, the point at which specificity exceeded 95% while achieving maximum sensitivity was selected as the alarm-based, workload-constrained cutoff for each model. However, because the threshold and range are also closely associated with the scoring scales of EWS models, further evaluation is necessary for broader applicability.

Continuous evaluation and adjustment of cutoff values based on clinical feedback and evolving practices are essential. These findings emphasize that cutoff selection should be guided not only by statistical performance but also by clinical usability, particularly in relation to alarm burden. Regular performance assessments of the model in real-world clinical settings will help maintain its effectiveness and relevance over time. Incorporating these recommendations into clinical practice may help healthcare providers leverage AI and ML models to predict and prevent clinical deterioration, potentially improving patient outcomes in future prospective implementations. This approach shifts the focus from traditional ROC-based evaluation methods to more practical metrics that align with clinical needs, emphasizing actionable insights and manageable alarm systems.

It is important to note that this study evaluated discriminative performance only and did not assess whether deployment of the alarm-based cutoff strategy improves patient outcomes, resource allocation, or clinician behaviour. Recent implementation studies demonstrate that real-world benefit from AI-based early warning systems depends on the integrated system as a whole—including the model, alert delivery mechanism, silencing rules, escalation pathways, and structured clinical response—rather than on discrimination performance alone [19,20,21]. Furthermore, the exclusion of patients with DNR orders and confirmed COVID-19 diagnoses, while intended to reduce confounding, may limit the generalizability of our findings and should be considered a potential source of selection bias. Prospective multi-centre implementation studies that capture the full care pathway will be necessary to confirm the clinical utility of the alarm-based threshold strategy described here.

The challenges of deploying AI-based monitoring systems extend beyond threshold selection and are broadly shared across cardiovascular digital health applications. Recent reviews of AI in cardiac rhythm monitoring have highlighted that false-positive alerts, limited external validation, and the absence of structured clinical response pathways remain key barriers to real-world implementation, irrespective of the underlying model architecture [22,23]. In atrial fibrillation detection, for instance, wearable-based AI systems demonstrate high sensitivity but generate substantial rates of clinically inconsequential alerts, underscoring that alarm burden management is not unique to general ward early warning systems but represents a fundamental challenge across AI-driven monitoring domains [22,23]. The alarm-based cutoff framework proposed in the present study—which explicitly constrains alert frequency to a clinically manageable range—directly addresses this cross-domain concern and may offer a generalizable design principle for threshold selection in AI monitoring systems more broadly. Nevertheless, as with other single-centre AI implementations, external validation across diverse hospital settings and patient populations remains necessary before the approach can be considered broadly applicable.

This study has several strengths. First, the large sample size and extended study period enhance statistical reliability. Second, we excluded COVID-19 patients to eliminate biases introduced by pandemic-related changes in critical care protocols, such as ICU admission delays and isolation practices. Additionally, DNR patients were excluded to reduce clinical heterogeneity and avoid confounding from end-of-life care decisions. These exclusions were intended to reduce clinical heterogeneity and improve the internal consistency of the analytic cohort.

However, this study has some limitations. It was conducted retrospectively at a single center, which may limit the generalizability of the findings. Further research involving multiple institutions and prospective validations is necessary to confirm these results. Moreover, this study focused on a specific AI model, and performance may vary with different algorithms and patient populations. Additionally, the primary outcome was a composite of unplanned ICU transfer (UIT, n = 524) and in-hospital cardiac arrest (IHCA, n = 182); as UIT accounts for the majority of events (74.2%), the reported performance may primarily reflect prediction of ICU transfer rather than cardiac arrest. Separate component-specific analyses are noted as a direction for future work. The alarm frequency metric was computed on a per-patient-day basis, counting at most one alarm per patient per calendar day; this convention caps the maximum possible alarm rate and should be considered when comparing with studies using raw time-point counts. Furthermore, the formal head-to-head comparison of discriminative performance was restricted to a common prediction time-point sample—time-points at which all three models could be simultaneously scored. To address the availability bias inherent in this restriction, alarm-based operating-point metrics (sensitivity, specificity, PPV, and alarm frequency) were additionally reported on this common sample; nevertheless, these estimates may still not fully reflect typical real-world deployment, where each model operates independently on its own available inputs. Patient-level analyses at the admission unit may therefore provide a more appropriate characterisation of real-world performance.

## 5. Conclusions

This study demonstrates the limitation of conventional ROC-based cutoff selection methods in real-world clinical settings, where alarm burden and workflow constraints play a critical role. Although traditional approaches optimize statistical performance, they often generate excessive or impractical alarm frequencies, limiting their clinical applicability. In contrast, the alarm-based cutoff strategy provides a more balanced approach by maintaining acceptable sensitivity and specificity while ensuring a manageable number of alarms. These findings emphasize that cutoff selection should be guided not only by statistical performance but also by clinical usability, particularly in relation to alarm burden. Incorporating clinically informed cutoff strategies may improve the practical implementation of AI-based early warning systems, potentially enhancing patient outcomes without overwhelming healthcare providers, pending prospective validation.

## Figures and Tables

**Table 1:** Baseline Characteristics of Study Population.

Group	Total	Non-CDE	CDE	*p*-Value	SMD
	(N = 48,790)	(N = 48,084)	(N = 706)		
Admission part				<0.001	
-Medical	28,840 (59.1%)	28,340 (58.9%)	500 (70.8%)		
-Surgical	19,950 (40.9%)	19,744 (41.1%)	206 (29.2%)		
Age (years)	60.0 [46.0; 73.0]	60.0 [46.0; 73.0]	76.0 [64.0; 82.0]	<0.001	0.797
Sex				<0.001	
-Male	22,606 (46.3%)	22,180 (46.1%)	426 (60.3%)		
-Female	26,184 (53.7%)	25,904 (53.9%)	280 (39.7%)		
Primary variables for MAES					
SBP (mmHg)	125.2 [116.2; 134.4]	125.3 [116.3; 134.4]	122.6 [113.3; 132.1]	<0.001	0.395
DBP (mmHg)	76.5 [71.2; 82.2]	76.6 [71.3; 82.2]	68.5 [63.1; 74.5]	<0.001	1.067
HR (beat/min)	75.5 [69.1; 82.6]	75.3 [69.0; 82.4]	88.4 [79.7; 97.4]	<0.001	1.004
RR (breaths/min)	18.5 [18.0; 19.2]	18.5 [18.0; 19.2]	19.3 [17.9; 21.4]	<0.001	0.497
Body temperature (°C)	36.8 [36.7; 37.0]	36.8 [36.7; 37.0]	36.8 [36.6; 37.0]	0.066	0.143
Additional variables for MAES					
Oxygen saturation (%)	98.5 [97.4; 99.3]	98.5 [97.4; 99.4]	98.3 [97.2; 98.9]	<0.001	0.384
Glasgow Coma Scale	15.0 [15.0; 15.0]	15.0 [15.0; 15.0]	14.0 [11.0; 15.0]	<0.001	0.990
Total bilirubin (mg/dL)	0.5 [0.3; 0.7]	0.5 [0.3; 0.7]	0.6 [0.4; 1.0]	<0.001	0.347
Lactate (mmol/L)	1.1 [0.8; 1.6]	1.0 [0.8; 1.6]	1.6 [1.0; 3.4]	<0.001	1.023
pH	7.4 [7.4; 7.4]	7.4 [7.4; 7.4]	7.4 [7.4; 7.5]	<0.001	0.340
Sodium (mEq/L)	139.0 [137.0; 141.0]	139.0 [137.0; 141.0]	138.0 [135.5; 140.5]	<0.001	0.072
Potassium (mEq/L)	4.1 [3.9; 4.4]	4.1 [3.9; 4.4]	4.0 [3.8; 4.3]	<0.001	0.189
Creatinine (mg/dL)	0.7 [0.6; 0.9]	0.7 [0.6; 0.9]	0.9 [0.6; 1.5]	<0.001	0.517
Hematocrit (%)	35.8 [31.4; 39.9]	35.9 [31.6; 39.9]	27.9 [25.8; 31.6]	<0.001	1.131
WBC (10^3^/μL)	7.3 [5.7; 9.4]	7.3 [5.7; 9.3]	9.9 [7.3; 12.9]	<0.001	0.517
Bicarbonate (mEq/L)	24.0 [22.0; 26.0]	23.9 [22.0; 25.8]	24.8 [21.0; 27.8]	<0.001	0.562
Platelet (10^3^/μL)	221.0 [176.0; 271.0]	222.0 [177.0; 272.0]	170.5 [96.5; 239.0]	<0.001	0.698
CRP (mg/dL)	8.8 [1.4; 35.0]	8.3 [1.3; 33.7]	46.9 [21.4; 83.5]	<0.001	0.914
MAES for each patient					
Median	2.6 [1.1; 5.6]	2.5 [1.1; 5.4]	14.2 [7.3; 26.6]	< 0.001	1.114
Min	0.8 [0.4; 1.6]	0.8 [0.4; 1.6]	3.4 [1.3; 8.8]	< 0.001	0.798
Max	9.1 [4.1; 19.1]	8.9 [4.0; 18.3]	59.8 [41.7; 77.1]	< 0.001	1.825
NEWS for each patient					
Median	1.0 [0.0; 2.0]	1.0 [0.0; 2.0]	3.0 [2.0; 5.0]	<0.001	1.162
Min	0.0 [0.0; 0.0]	0.0 [0.0; 0.0]	0.0 [0.0; 2.0]	<0.001	0.798
Max	4.0 [2.0; 5.0]	4.0 [2.0; 5.0]	8.0 [6.0; 11.0]	<0.001	1.128
MEWS for each patient					
Median	1.0 [1.0; 1.0]	1.0 [1.0; 1.0]	2.0 [1.0; 2.0]	<0.001	0.860
Min	1.0 [0.0; 1.0]	1.0 [0.0; 1.0]	1.0 [0.0; 1.0]	0.038	0.009
Max	2.0 [1.0; 3.0]	2.0 [1.0; 3.0]	4.0 [3.0; 6.0]	<0.001	1.097

Abbreviations: CDE, Clinical Deterioration Event; SBP, Systolic Blood Pressure; DBP, Diastolic Blood Pressure; HR, Heart rate; RR, Respiratory Rate; CRP, C-reactive protein; WBC, White Blood Cell; MAES, Major Adverse Event Score; NEWS, National Early Warning Score; MEWS, Modified Early Warning Score.

**Table 2:** Comparison of cutoff values, sensitivity, specificity, and alarm frequency across different cutoff selection methods for VC-MAES, NEWS, and MEWS. Cutoff values derived from conventional statistical methods (Youden index, index of union, Euclidean distance, and F1 score) are presented alongside the alarm-based cutoff values. While ROC-based methods yield higher sensitivity, they are associated with substantially increased alarm frequencies, exceeding clinically acceptable thresholds. In contrast, the alarm-based cutoff approach achieves a balance between sensitivity and specificity while maintaining an acceptable number of alarms per 100 patient-days.

Index	Score	Cutoff Value	Sensitivity	Specificity	PPV (%)	Alarms Per 100 Patient-Days
Youden Index	MAES	15	0.7825	0.8429	1.19	19.2
	NEWS	4	0.7137	0.7943	0.41	23.3
	MEWS	2	0.7330	0.7313	0.31	37.6
IU	MAES	15	0.7825	0.8429	1.19	19.2
	NEWS	4	0.7137	0.7943	0.41	23.3
	MEWS	2	0.7330	0.7313	0.31	37.6
ED	MAES	14	0.7969	0.8272	1.11	21.1
	NEWS	4	0.7137	0.7943	0.41	23.3
	MEWS	2	0.7330	0.7313	0.31	37.6
F1 score	MAES	60	0.1938	0.9970	13.59	0.7
	NEWS	11	0.0721	0.9970	2.76	0.7
	MEWS	6	0.0826	0.9963	2.42	0.8
Optimal Alarm-based Cutoff value	MAES	29	0.5853	0.9526	2.91	6.4
	NEWS	7	0.3775	0.9510	0.90	6.3
	MEWS	4	0.3049	0.9687	1.08	5.2

Abbreviations: IU, Index of Union; ED, Euclidean Distance; MAES, Major Adverse Event Score; NEWS, National Early Warning Score; MEWS, Modified Early Warning Score. Operating-point metrics (sensitivity, specificity, PPV, and alarm frequency) for the alarm-based cutoff row are computed on each model’s own full evaluable sample (VC-MAES: n = 7425 events; NEWS: n = 3494 events; MEWS: n = 3198 events). For direct cross-model comparison on identical observations, the same metrics recomputed on the common prediction time-point sample (n = 1678 events) are reported in Supplementary Table S6.

**Table 3:** Comparison of time to first prediction and diagnostic performance across different cutoff selection methods for VC-MAES, NEWS, and MEWS. Time to first prediction represents the interval between the first threshold crossing and the occurrence of clinical deterioration events. Although conventional methods such as Youden index and Euclidean distance provide earlier predictions, they are associated with excessive alarm rates. The alarm-based cutoff approach achieves a clinically acceptable balance by maintaining reasonable prediction timing while reducing unnecessary alarms.

Index	Score	Mean Time to First Prediction (min)	95% CI	Sensitivity	Specificity
Youden Index	MAES	255	254.5–257.1	0.7825	0.8429
	NEWS	260.4	258.7–262.0	0.7137	0.7943
	MEWS	254.7	252.9–256.4	0.7330	0.7313
IU	MAES	255	254.5–257.1	0.7825	0.8429
	NEWS	260.4	258.7–262.0	0.7137	0.7943
	MEWS	254.7	252.9–256.4	0.7330	0.7313
ED	MAES	258	257.1–259.6	0.7969	0.8272
	NEWS	260.4	258.7–262.0	0.7137	0.7943
	MEWS	254.7	252.9–256.4	0.7330	0.7313
F1 score	MAES	147	145.7–148.5	0.1938	0.9970
	NEWS	221.4	219.6–223.2	0.0721	0.9970
	MEWS	202.5	200.4–204.5	0.0826	0.9963
Optimal Alarm-based Cutoff value	MAES	219	218.3–221.2	0.5853	0.9526
	NEWS	241.5	239.7–243.3	0.3775	0.9510
	MEWS	223.4	221.4–225.3	0.3049	0.9687

Abbreviations: IU, Index of Union; ED, Euclidean Distance; MAES, Major Adverse Event Score; NEWS, National Early Warning Score; MEWS, Modified Early Warning Score.

## Data Availability

The datasets generated and/or analyzed during the current study are not publicly available due to patient privacy; however, they are available from the corresponding author upon reasonable request.

## Supplementary Materials

The following supporting information can be downloaded at: https://www.mdpi.com/article/10.3390/jcm15145753/s1, Table S1: Detailed cutoff values, sensitivity, specificity, and alarm frequencies for VC-MAES across all thresholds. Table S2: Detailed cutoff values, sensitivity, specificity, and alarm frequencies for NEWS across all thresholds. Table S3: Detailed cutoff values, sensitivity, specificity, and alarm frequencies for MEWS across all thresholds. Table S4: AUROC and AUPRC with 95% confidence intervals (observation-level and patient-level) for VC-MAES, NEWS, and MEWS across prediction horizons of 6, 8, 12, and 24 h. Table S5: Variable-specific missingness proportions and mean input intervals for all predictor variables used by VC-MAES, NEWS, and MEWS. Table S6: Alarm-based cutoff performance (sensitivity, specificity, PPV, and alarm frequency) for VC-MAES, NEWS, and MEWS computed on the common prediction time-point sample (674,560 time-points; 1678 events), enabling a direct head-to-head comparison of operating-point metrics across all three models on identical observations. Figure S1: Calibration plot of VC-MAES at the 6-h prediction horizon. The solid line represents the observed calibration curve; the dashed line represents ideal calibration. The calibration plot reveals overestimation at low predicted probabilities and imperfect calibration in the upper predicted-risk range (predicted probability >0.4), consistent with a discrimination-optimised model applied in a low-prevalence setting (~1.4%). Formal recalibration would be required before using the model output as a direct probability estimate.

## Abbreviations

The following abbreviations are used in this manuscript:

AUROC	Area under the ROC curve
CDE	Clinical deterioration event
CRP	C-reactive protein
DNR	Do-not-resuscitate
EWS	Early warning score
EMR	Electronic medical record
ED	Euclidean distance
IU	Index of union
IHCA	In-hospital cardiac arrest
ICU	Intensive care unit
IQR	Interquartile range
ML	Machine learning
MAES	Major adverse event score
MEWS	Modified early warning score
NEWS	National early warning score
ROC	Receiver operating characteristic
UIT	Unplanned ICU transfer
VC-MAES	VitalCare-major adverse event score
