# Supplementary figures and images for: Determining a Clinically Applicable Cutoff in AI Algorithms for Predicting Clinical Deterioration: A Workload-Constrained, Alarm-Based Approach
Source: J Clin Med. 2026 Jul 22;15(14):5753. doi: 10.3390/jcm15145753 (PMC13413000; doi:10.3390/jcm15145753)

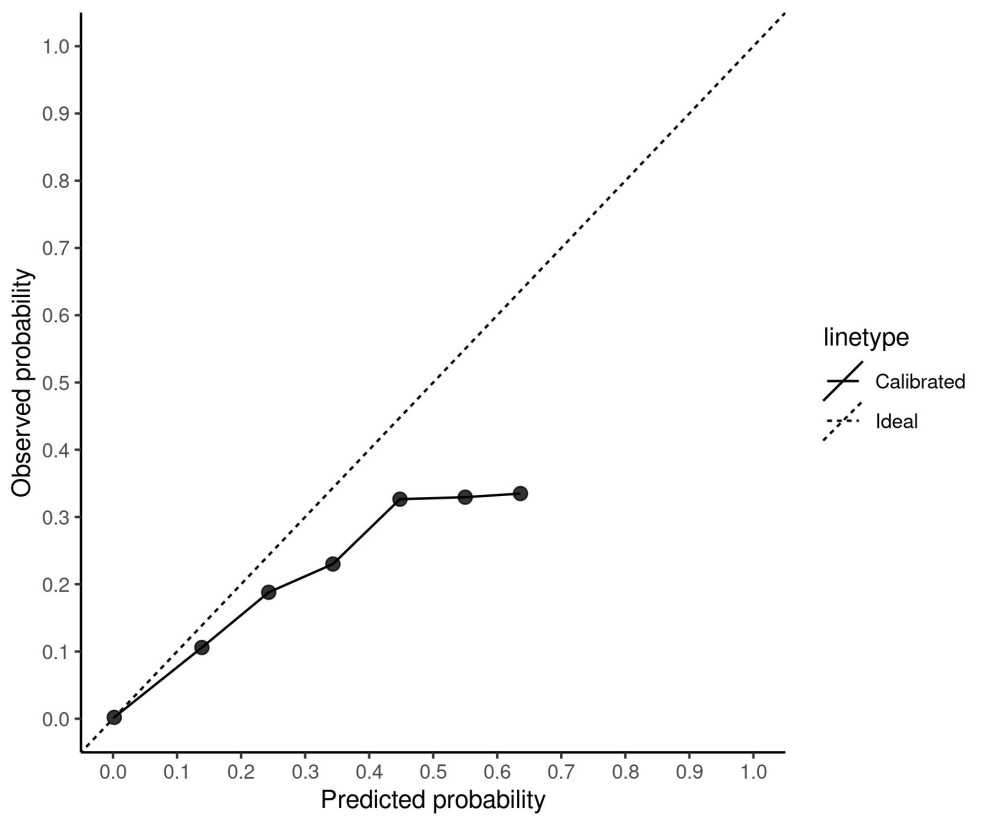

Supplement: Supplementary file 1 [file jcm-15-05753-s001.zip › Figure_S1.jpg]
